# Estimated pulse wave velocity associated with cognitive phenotypes in a rural older population in China: A cohort study

**DOI:** 10.1002/alz.14491

**Published:** 2025-01-17

**Authors:** Jiahui Li, Yifei Ren, Lidan Wang, Xinrui Zou, Xueran Ding, Tingting Hou, Qinghua Zhang, Shi Tang, Xiaojuan Han, Lin Song, Yajun Liang, Yongxiang Wang, Lin Cong, Yifeng Du, Chengxuan Qiu

**Affiliations:** ^1^ Department of Neurology Shandong Provincial Hospital affiliated to Shandong First Medical University Jinan Shandong P. R. China; ^2^ Department of Neurology Shandong Provincial Hospital Shandong University Jinan Shandong P. R. China; ^3^ Shandong Provincial Clinical Research Center for Geriatric Neurological Diseases Jinan Shandong P. R. China; ^4^ Department of Global Public Health Karolinska Institutet Stockholm Sweden; ^5^ Institute of Brain Science and Brain‐Inspired Research Shandong First Medical University & Shandong Academy of Medical Sciences Jinan Shandong P. R. China; ^6^ Aging Research Center Department of Neurobiology Care Sciences and Society, Karolinska Institutet‐Stockholm University Solna Sweden; ^7^ Medical Science and Technology Innovation Center Shandong First Medical University & Shandong Academy of Medical Sciences Jinan Shandong P. R. China

**Keywords:** Alzheimer's disease, cognitive function, cohort study, dementia, estimated pulse wave velocity, vascular dementia

## Abstract

**INTRODUCTION:**

To examine the longitudinal association between estimated pulse wave velocity (ePWV) and cognitive phenotypes in a rural Chinese older population.

**METHODS:**

This population‐based study included 1857 dementia‐free participants (age ≥60 years) who were examined in 2014 and followed in 2018. ePWV was calculated using age and mean blood pressure (MBP). Cognitive function was assessed using the Mini‐Mental State Examination (MMSE) and neuropsychological tests. Dementia was diagnosed following the Diagnostic and Statistical Manual of Mental Disorders, Fourth Edition (DSM‐IV) criteria. Data were analyzed using Cox proportional‐hazards models, linear regression models, and restricted cubic spline (RCS) curves.

**RESULTS:**

Per 1‐m/s increase in ePWV was associated with an adjusted hazard ratio (HR) (95% confidence interval [CI]) of 1.51(1.30–1.75) for dementia and 1.58(1.33–1.89) for Alzheimer's disease (AD), and with an MMSE score decline (adjusted *β*‐coefficient = −0.36; 95% CI = −0.52 to −0.21). A nonlinear association was observed between baseline ePWV and follow‐up cognitive scores.

**DISCUSSION:**

A higher ePWV is associated with an increased risk of dementia, AD, accelerated cognitive decline, and poorer cognitive performance in older adults.

**Highlights:**

- An increased estimated pulse wave velocity (ePWV) was associated with incident dementia, Alzheimer's disease, and accelerated cognitive decline in a rural Chinese older population.
- A higher ePWV at baseline was associated with lower scores of global cognition and multiple cognitive domains at the 4‐year follow‐up.
- An increased ePWV may be a risk factor for dementia and accelerated cognitive deterioration in aging.

## 1 BACKGROUND

The walls of large arteries lose elasticity over time with aging, which leads to increased arterial stiffness.^1^ Central arterial stiffening can facilitate the transfer of high pressure and flow pulsatility to high‐flow organs to penetrate deeper into the microcirculation, resulting in microvascular damage. The brain as a high‐flow organ is susceptible to the adverse effects of aortic stiffening.^2^ Evidence has accumulated that increased arterial stiffness is associated with cognitive phenotypes ranging from poor or impaired cognitive function,^3^, ^4^, ^5^, ^6^ accelerated cognitive decline,^7^, ^8^, ^9^, ^10^, ^11^ and an increased risk of dementia.^12^, ^13^, ^14^

Carotid‐femoral pulse wave velocity (cfPWV) is regarded as a simple, reproducible, and non‐invasive measurement for arterial stiffness.^15^ However, measurement of cfPWV requires expensive devices and professional technical staff, which is unfeasible to be applied in large‐scale studies of the general population settings, especially in remote rural areas. Thus, the estimated pulse wave velocity (ePWV), calculated simply from age and mean blood pressure (MBP), was developed to assess the degree of arterial stiffness. The Reference Values for Arterial Stiffness Collaboration has developed an algorithm to estimate ePWV,^16^ which has shown a predictive value similar to that of cfPWV for the combined cardiovascular endpoint of cardiovascular death, nonfatal myocardial infarction, stroke, and hospitalization for ischemic heart disease.^17^ Indeed, several studies have linked increased ePWV with elevated risks of cardiovascular and cerebrovascular events and mortality.^18^, ^19^, ^20^, ^21^ However, evidence linking ePWV with cognitive phenotypes in old age remains limited. Data from the Northern Manhattan Study recently showed that increased ePWV was associated with poor cognition and accelerated cognitive decline, which underscores the role of arterial stiffness in cognitive aging.^22^ In this population‐based cohort study, we sought to investigate the associations of ePWV with incident dementia, Alzheimer's disease (AD), vascular dementia (VaD), cognitive decline, and function of various cognitive domains in a rural Chinese older population.

## 2 METHODS

### 2.1 Study populations

This is a population‐based cohort study. The study participants were drawn from the Shandong Yanggu Study of Aging and Dementia (SYS‐AD), which targeted rural older residents who were living in Yanlou Town, Yanggu County, western Shandong Province, as reported previously.^23^ In brief, a total of 3274 participants who were 60 years of age and older undertook the baseline examination in August to December 2014. Survivors of the SYS‐AD baseline participants were invited for a follow‐up examination that was conducted in March to September 2018, as part of the baseline assessments of the Multimodal Interventions to Delay Dementia and Disability in Rural China (MIND‐China), as fully described elsewhere.^24^, ^25^

**FIGURE 1 alz14491-fig-0001:**
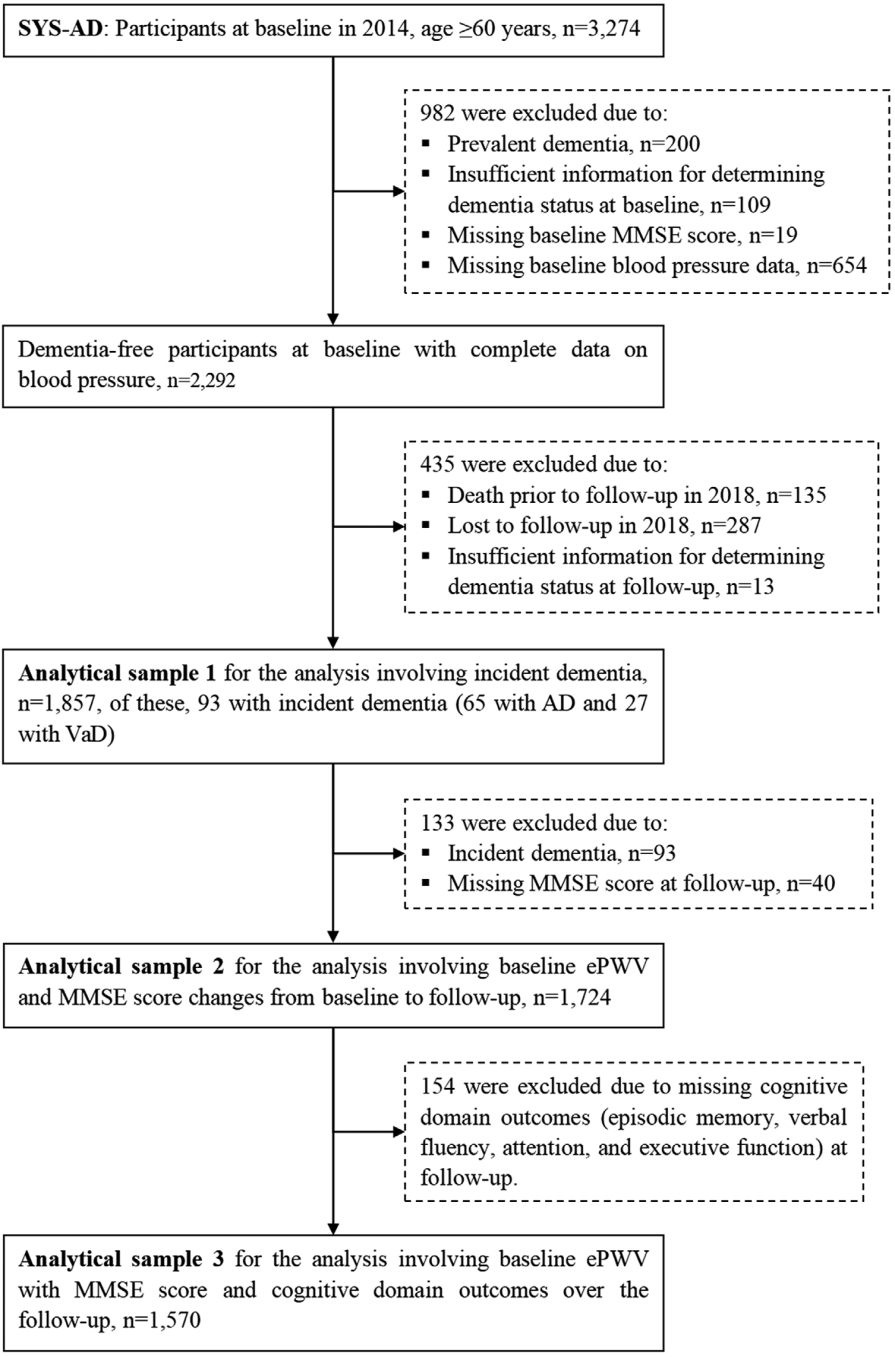
Flowchart of the study participants in SYS‐AD, 2014–2018. AD, Alzheimer's disease; ePWV, estimated pulse wave velocity; MMSE, Mini‐Mental State Examination; SYS‐AD, Shandong Yanggu Study of Aging and Dementia; VaD, vascular dementia.

Out of the 3274 participants at baseline, 982 were excluded due to prevalent dementia that was diagnosed according to the Diagnostic and Statistical Manual of Mental Disorders, Fourth Edition (DSM‐IV) criteria (*n* = 200), insufficient information for determining dementia status (*n* = 109), missing data on the Mini‐Mental State Examination (MMSE) (*n* = 19), or missing data on blood pressure measurements (*n* = 654) at baseline. Of the remaining 2292 dementia‐free participants at baseline with complete data on blood pressure measurements, we further excluded 435 persons due to death prior to the follow‐up examination in 2018 (*n* = 135), being lost to follow‐up (*n* = 287), or insufficient information for determining the dementia status at follow‐up (*n* = 13). Thus, 1857 participants were included to examine the association between baseline ePWV and incident dementia (analytical sample 1). Overall, compared with persons who were excluded from the analysis, participants in the analytical sample were relatively younger (mean age 70.7 vs 74.3 years, *p* < 0.001), more likely to be women (60.0% vs 47.6%, *p* < 0.001), less likely to receive no school education (38.6% vs 47.6%, *p* = 0.002), and had slightly lower ePWV (12.27 vs 12.98 m/s, *p* < 0.001). Of the 1857 participants, we further excluded 133 persons who were diagnosed with incident dementia at follow‐up in 2018 (*n* = 93) or who had missing data on MMSE score at follow‐up (*n* = 40), leaving 1724 participants for the analysis on the association between baseline ePWV and changes in MMSE score from baseline to follow‐up (follow‐up MMSE score minus baseline MMSE score) (analytical sample 2). Among the 1724 participants, we additionally excluded 154 persons due to missing data on various cognitive domains (i.e., episodic memory, verbal fluency, attention, and executive function) that were assessed using the neuropsychological test battery at follow‐up. Thus, the analytical sample for examining the association of baseline ePWV with MMSE score and cognitive domain outcomes at follow‐up consisted of 1570 participants (analytical sample 3). The flowchart of study participants is illustrated in Figure 1.

**RESEARCH IN CONTEXT**

1. **Systematic review**: We searched PubMed for relevant literature. Although current evidence has linked increased arterial stiffness with cognitive impairment and dementia, the relationship between ePWV (a measure of arterial stiffness) and cognitive phenotypes remains largely unexplored, especially among Asian populations.
2. **Interpretation**: This population‐based prospective cohort study showed that a greater ePWV was associated with an increased risk of incident dementia, Alzheimer's disease, and accelerated global cognitive decline, and further revealed that a greater ePWV at baseline was associated with lower scores of global cognition and multiple cognitive domains at the 4‐year follow‐up. These findings highlight the potential that an increased ePWV may be a risk factor for dementia and accelerated cognitive deterioration in a rural Chinese older population.
3. **Future directions**: Future large‐scale long‐term prospective cohort studies are warranted to clarify the potential causal relationship between ePWV and cognitive phenotypes as well as the neuropathological mechanisms underlying the relationship.

The SYS‐AD and the MIND‐China study were approved by the ethics committee at Shandong Provincial Hospital affiliated with Shandong First Medical University in Jinan, Shandong Province. Written informed consent was obtained from all participants, or in the case of cognitively impaired persons, from a proxy (usually a guardian or a family member). The MIND‐China study was registered in the Chinese Clinical Trial Registry (registration no.: ChiCTR1800017758).[Fig alz14491-fig-0001]

### 2.2 Data collection

At baseline, trained medical staff collected data through face‐to‐face interviews, clinical examinations, cognitive testing, and laboratory tests following a structured questionnaire, as reported previously.^23^, ^26^ The questionnaire included information on demographic factors (e.g., sex, age, and education), lifestyle factors (e.g., smoking and alcohol consumption), health conditions (e.g., hypertension, diabetes, hyperlipidemia, coronary heart disease, and stroke), and genetic factors (e.g., apolipoprotein E [*APOE*] genotype). The survival status of all participants at the follow‐up was determined via linkage with death records or interviews with the local village doctors.

### 2.3 Calculation of ePWV

Arterial blood pressure was measured on the participant's right arm in a sitting position after a 5‐min rest using an electronic blood pressure monitor, with the cuff being maintained at the heart level. The ePWV was calculated using the following formula that was developed by the Reference Values for Arterial Stiffness Collaboration^16^ and described by Greve and colleagues:^17^

ePWV = 9.587 − (0.402 × age) + (4.560 × 10^−3^ × age^2^) − (2.621 × 10^−5^ × age^2^ × MBP) + (3.176 × 10^−3^ × age × MBP) − (1.832 × 10^−2^ × MBP).

MBP was determined as diastolic BP + 0.4 (systolic BP − diastolic BP). ePWV could be calculated using either the normal population or the reference population, depending on the individual's cardiovascular risk factor burden.^17^ Because the large majority of participants in the SYS‐AD and the MIND‐China cohorts had at least one cardiovascular risk factor, ePWV was calculated using the equation derived from the reference population. We categorized ePWV into three groups according to tertiles of the ePWV distribution: lower tertile (ePWV ≤11.65), medium tertile (11.65 < ePWV ≤ 12.71), and upper tertile (ePWV >12.71).

### 2.4 Assessment of cognitive function

We assessed the following cognitive phenotypes, that is, global cognitive function, changes in global cognition, function of multiple cognitive domains (e.g., episodic memory, verbal fluency, attention, and executive function), dementia, and subtypes of dementia (e.g., AD and VaD).

At both baseline (2014) and follow‐up (2018) examinations, global cognitive function was assessed using the validated Chinese version of the MMSE.^27^ At follow‐up in 2018, a neuropsychological test battery was used to assess the function of four cognitive domains, that is, episodic memory (Auditory Verbal Learning Test [AVLT] immediate recall, long‐delayed free recall, and long‐delayed recognition),^28^ verbal fluency (Verbal Fluency Test categories of animals, fruits, and vegetables),^29^ attention (Digit Span Test [DST] forward^30^ and Trail Making Test [TMT] Part A^31^), and executive function (DST backward^30^ and TMT B^31^). The raw score of each of the tests was standardized into a *z*‐score using the mean and standard deviation (SD) derived from all the study participants who were free of dementia. Because all cognitive domains were assessed using more than one test, we created a composite *z*‐score for each of the cognitive domains by averaging the *z*‐scores of the tests for that domain. The global cognitive function was assessed by averaging the *z*‐scores of all four domains.

### 2.5 Clinical diagnosis of dementia, AD, and VaD

At both baseline and follow‐up examinations, dementia was clinically diagnosed according to the DSM‐IV criteria, ^32^ following a three‐step diagnostic procedure, as reported previously.^23^, ^25^ In brief, trained medical staff first conducted the face‐to‐face interviews, clinical examinations, and assessments of cognitive and physical functioning, and recorded all information following structured questionnaires. Then the neurologists reviewed all the records to screen participants who were suspected to have dementia or who had insufficient information for determining the dementia status for further evaluation. Finally, senior neurologists conducted additional face‐to‐face interviews with participants who were suspected to have dementia and with their caregivers. During the interview, the neurologists reassessed their medical history, cognitive status, daily living ability, and, whenever available, neuroimaging data, and made a final diagnosis of dementia following the DSM‐IV criteria.^32^ Dementia was further classified into AD according to the National Institute on Aging–Alzheimer's Association (NIA‐AA) criteria for probable AD dementia^33^ and VaD following the National Institute of Neurological Disorders and Stroke and the Association Internationale pour la Recherche et l'Enseignement en Neurosciences (NINDS‐AIREN) criteria for probable VaD.^34^

### 2.6 Assessment of covariates

At baseline, weight and height were measured in light clothes without shoes. Body mass index (BMI) was calculated as measured weight in kilograms divided by height in meters squared. After an overnight fast, peripheral blood samples were taken, and fasting blood glucose and serum lipids were measured in the local clinical laboratories of Yanlou Town Hospital following standard protocols. *APOE* genotype was detected using multiple polymerase chain reaction (PCR) amplification (iGeneTech Bioscience Co., Ltd., Beijing, China) and was dichotomized into carriers versus noncarriers of the ε4 allele.

Educational level was categorized as receiving no formal school education, primary school, and middle school or above. Smoking status and alcohol consumption were categorized as never or ever smoking or alcohol drinking, respectively. Hypertension was defined as having blood pressure ≥140/90 mmHg or the use of any antihypertensive agents. Diabetes was defined as fasting blood glucose ≥7.0 mmol/L use of antidiabetic agents, or having a self‐reported history of diabetes. Dyslipidemia was defined as total serum cholesterol ≥6.22 mmol/L, triglyceride ≥2.27 mmol/L, low‐density lipoprotein cholesterol ≥4.14 mmol/L, or high‐density lipoprotein cholesterol <1.04 mmol/L, or use of hypolipidemic agents. Coronary heart disease was defined according to self‐reported history or electrocardiography examination, which included angina pectoris, myocardial infarction, and coronary intervention. Stroke was ascertained according to self‐reported history of stroke or neurological examination.

### 2.7 Statistical analysis

One‐way analysis of variance (ANOVA) was used to test differences for continuous variables, and the χ^2^ test was used to test differences for categorical variables. We used the Cox proportional‐hazards models to examine the association of baseline ePWV with incident dementia, AD, and VaD diagnosed at follow‐up. We fitted two regression models while controlling for different potential confounders. Model 1 was adjusted for sex and education. Model 2 was further adjusted for BMI, *APOE* genotype, smoking, alcohol consumption, use of antihypertensive medication, diabetes mellitus, hyperlipidemia, coronary heart disease, and stroke.

General linear regression models were used to examine the association between baseline ePWV and changes in MMSE score from baseline to follow‐up examinations among participants who were free of dementia at both baseline and follow‐up. In the linear regression models, we additionally adjusted for baseline MMSE score and follow‐up time. In order to achieve an excellent model fit while ensuring the interpretability of the model, we used the restricted cubic spline (RCS) curves with three knots at the 10th, 50th, and 90th percentiles to flexibly model the nonlinear association of baseline ePWV with MMSE score and *z*‐scores of global cognition and various cognitive domains measured at follow‐up.^35^ According to the RCS results, we further used the linear regression models to estimate *β* coefficients (95% confidence intervals [CIs]) of MMSE score and *z*‐scores of cognitive domains associated with ePWV stratified by the inflection point of ePWV.

IBM SPSS Statistics for Windows, version 26.0 (Armonk, NY: IBM Corp) was used for all statistical analyses, except the RCS analysis, where the R package for Windows (version 4.3.1, R Foundation for Statistical Computing, Vienna, Austria) was used. A two‐tailed *p* < 0.05 was considered statistically significant, and we used the Bonferroni test to adjust for multiple comparisons.

## 3 RESULTS

### 3.1 Baseline characteristics of study participants

At baseline, the mean age of the 1857 participants was 70.70 (SD = 4.69) years, 60.0% were women, and 38.6% had received no formal schooling (Table 1). Compared to participants with lower and medium ePWVs, those with upper ePWV were older, less educated, less likely to smoke, and more likely to be female and to have a history of hypertension and a lower MMSE score. In addition, participants with medium and upper ePWVs were more likely to have hypercholesterolemia and a higher BMI than those with lower ePWV (*p* < 0.05). There were no significant differences among the three groups in the distribution of *APOE* genotype, diabetes, coronary heart disease, and stroke (*p* > 0.05) (Table 1).

**TABLE 1:** Characteristics of study participants at baseline (*n* = 1857).

		ePWV (m/s) at baseline, tertiles			
Characteristics	Total sample	Lower (ePWV≤11.65)	Medium (11.65＜ePWV≤12.71)	Upper (ePWV> 12.71)	*p*‐value
Number of participants	1857	622	616	619	–
Age, years, mean (SD)	70.70 (4.69)	67.74 (2.21)	69.60 (3.11)	74.75 (5.01)	<0.001
Women, *n* (%)	1115 (60.0)	358 (57.6)	359 (58.3)	398 (64.3)	0.029
Educational level, *n* (%)					<0.001
No formal schooling	716 (38.6)	198 (31.8)	207 (33.6)	311 (50.2)	
Primary school	856 (46.1)	320 (51.4)	301 (48.9)	235 (38.0)	
Middle school or above	285 (15.3)	104 (16.7)	108 (17.5)	73 (11.8)	
*APOE* ε4 allele, *n* (%)^a^	266 (14.3)	90 (14.5)	83 (13.5)	93 (15.0)	0.595
Ever smoking, *n* (%)	630 (33.9)	234 (37.6)	208 (33.8)	188 (30.4)	0.026
Alcohol consumption, *n* (%)	641 (34.5)	225 (36.2)	228 (37.0)	188 (30.4)	0.028
BMI, kg/m^2^, mean (SD)	25.07 (3.82)	24.57 (3.68)	25.45 (3.87)	25.20 (3.85)	<0.001
Hypertension, *n* (%)	1460 (78.6)	376 (60.5)	514 (83.4)	570 (92.1)	<0.001
Use of antihypertensive medication, *n* (%)	615 (33.1)	122 (19.6)	236 (38.3)	257 (41.5)	<0.001
Diabetes, *n* (%)	277 (14.9)	95 (15.3)	101 (16.4)	81 (13.1)	0.252
Hyperlipidemia, *n* (%)	558 (30.0)	154 (24.8)	203 (33.0)	201 (32.5)	0.002
Coronary heart disease, *n* (%)	348 (18.7)	101 (16.2)	122 (19.8)	125 (20.2)	0.144
Stroke, *n* (%)	162 (8.7)	48 (7.7)	59 (9.6)	55 (8.9)	0.502
SBP, mmHg, mean (SD)	145.02 (19.24)	130.66 (12.56)	145.55 (13.01)	158.94 (19.57)	<0.001
DBP, mmHg, mean (SD)	86.65 (10.03)	79.46 (7.46)	88.92 (8.39)	91.61 (9.71)	<0.001
MBP, mmHg, mean (SD)	110.00 (11.91)	99.94 (7.88)	111.57 (7.60)	118.54 (11.35)	<0.001
ePWV, m/s, mean (SD)	12.27 (1.27)	10.94 (0.55)	12.18 (0.29)	13.69 (0.83)	–
MMSE score, mean (SD)	21.94 (5.35)	23.05 (4.76)	22.46 (5.45)	20.30 (5.41)	<0.001

Abbreviations: *APOE*, apolipoprotein E gene; BMI, body mass index; DBP: diastolic blood pressure; ePWV, estimated pulse wave velocity; MBP, mean blood pressure; MMSE, Mini‐Mental State Examination; SBP, systolic blood pressure; SD, standard deviation.

^a^ The number of participants with missing values was 76 for the *APOE* genotype. In subsequent analyses, a dummy variable was created for participants with missing data as the covariate.

### 3.2 Associations of baseline ePWV with incident dementia, AD, and VaD at follow‐up (analytical sample 1, *n* = 1857)

During an average of 3.5 years of follow‐up, 93 individuals developed dementia, including 65 with AD and 27 with VaD. As a continuous variable, every 1‐m/s increment in ePWV was associated with a multivariable‐adjusted hazard ratio (HR) of 1.51 (95% CI: 1.30–1.75) for incident dementia, 1.58 (1.33–1.89) for AD, and 1.30 (0.96–1.77) for VaD. When ePWV was categorized into tertiles, the upper tertile (vs lower tertile) of ePWV was associated with multivariable‐adjusted HR of 3.25 (1.78–5.96) for incident dementia and 3.92 (1.86–8.22) for AD. In addition, the medium tertile (vs lower tertile) of ePWV was associated with multivariable‐adjusted HR of 3.94 (1.11–14.03) for VaD. After the Bonferroni correction, the association between ePWV and VaD became statistically nonsignificant (Table 2).

**TABLE 2:** Associations of baseline ePWV with incident dementia, AD, and VaD (*n* = 1857).

ePWV	No. of participants	No. of cases	Model 1		Model 2	
			HR (95% CI)	*p*‐value	HR (95% CI)	*p*‐value
**All‐cause dementia**						
ePWV, continuous variable						
(each 1 m/s increment)	1857	93	1.50 (1.29–1.73)	<0.001^*^	1.51 (1.30–1.75)	<0.001^*^
ePWV, categorical variable (tertiles)						
Lower	622	14	1.00 (reference)	–	1.00 (reference)	–
Medium	616	27	1.89 (0.99–3.61)	0.053	1.90 (0.99–3.64)	0.053
Upper	619	52	3.24 (1.79–5.87)	<0.001^*^	3.25 (1.78–5.96)	<0.001^*^
*p* for linear trend			<0.001		<0.001	
**Alzheimer's disease**						
ePWV, continuous variable						
(each 1 m/s increment)	1829	65	1.59 (1.34–1.88)	<0.001^*^	1.58 (1.33–1.89)	<0.001^*^
ePWV, categorical variable (tertiles)						
Lower	617	9	1.00 (reference)	–	1.00 (reference)	–
Medium	604	16	1.74 (0.77–3.95)	0.183	1.81 (0.79–4.12)	0.159
Upper	608	40	3.82 (1.85–7.92)	<0.001^*^	3.92 (1.86–8.22)	<0.001^*^
*p* for linear trend			<0.001		<0.001	
**Vascular dementia**						
ePWV, continuous variable						
(each 1 m/s increment)	1791	27	1.31 (0.98–1.73)	0.066	1.30 (0.96–1.77)	0.093
ePWV, categorical variable (tertiles)						
Lower	600	3	1.00 (reference)	–	1.00 (reference)	–
Medium	598	13	4.36 (1.24–15.31)	0.022^*^	3.94 (1.11–14.03)	0.034
Upper	593	11	3.37 (0.93–12.17)	0.063	2.85 (0.78–10.41)	0.114
*p* for linear trend			0.095		0.190	

*Note*: Model 1 was adjusted for sex and education. Model 2 was adjusted for sex, education, BMI, *APOE* genotype, smoking, alcohol consumption, use of antihypertensive medication, diabetes mellitus, hyperlipidemia, coronary heart disease, and stroke.

Abbreviations: AD, Alzheimer's disease; *APOE*, apolipoprotein E gene; BMI, body mass index; CI, confidence interval; ePWV, estimated pulse wave velocity; HR, hazard ratio; VaD, vascular dementia.

* The significant associations survived the Bonferroni corrections for multiple comparisons.

### 3.3 Association of baseline ePWV with cognitive decline from baseline to follow‐up (analytical sample 2, *n* = 1724)

Among individuals who were free of dementia at both baseline and follow‐up, an increased ePWV at baseline was significantly associated with a greater decline in the MMSE score (*β* = −0.36; 95% CI = −0.52 to −0.21) after adjustment for all confounding variables. When ePWV was analyzed as tertiles, the upper tertile of ePWV was significantly associated with a greater decline in the MMSE score (*β* = −0.83; 95% CI = −1.29 to −0.38) compared to the lower tertile of ePWV (Table 3).

**TABLE 3:** Associations of baseline ePWV with MMSE score changes from baseline to follow‐up among dementia‐free participants both at baseline and follow‐up examinations (*n* = 1724).

	Model 1		Model 2	
ePWV	*β* coefficient (95% CI)	*p*‐value	*β* coefficient (95% CI)	*p*‐value
Continuous variables (each 1 m/s increment)	−0.35 (−0.50 to −0.20)	<0.001	−0.36 (−0.52 to −0.21)	<0.001
Categorical variable (tertiles)				
Lower	0.00 (reference)	–	0.00 (reference)	–
Medium	−0.22 (−0.66 to 0.21)	0.319	−0.22 (−0.67 to 0.22)	0.321
Upper	−0.84 (−1.28 to −0.40)	<0.001	−0.83 (−1.29 to −0.38)	<0.001
*p* for linear trend	<0.001		<0.001	

*Note*: *β* coefficients (95% CIs) in model 1 and model 2 represent average changes in MMSE score from the baseline examination in 2014 to the follow‐up examination in 2018. Model 1 was adjusted for sex, education, baseline MMSE score, and follow‐up time (years). Model 2 was adjusted for sex, education, baseline MMSE score, follow‐up time (years), BMI, *APOE* genotype, smoking, alcohol consumption, use of antihypertensive medication, diabetes mellitus, hyperlipidemia, coronary heart disease, and stroke.

Abbreviations: *APOE*, apolipoprotein E gene; BMI, body mass index; CI, confidence interval; ePWV, estimated pulse wave velocity; MMSE, Mini‐Mental State Examination.

### 3.4 Association of baseline ePWV with global and domain‐specific cognitive function assessed at follow‐up (analytical sample 3, *n* = 1570)

**FIGURE 2:**
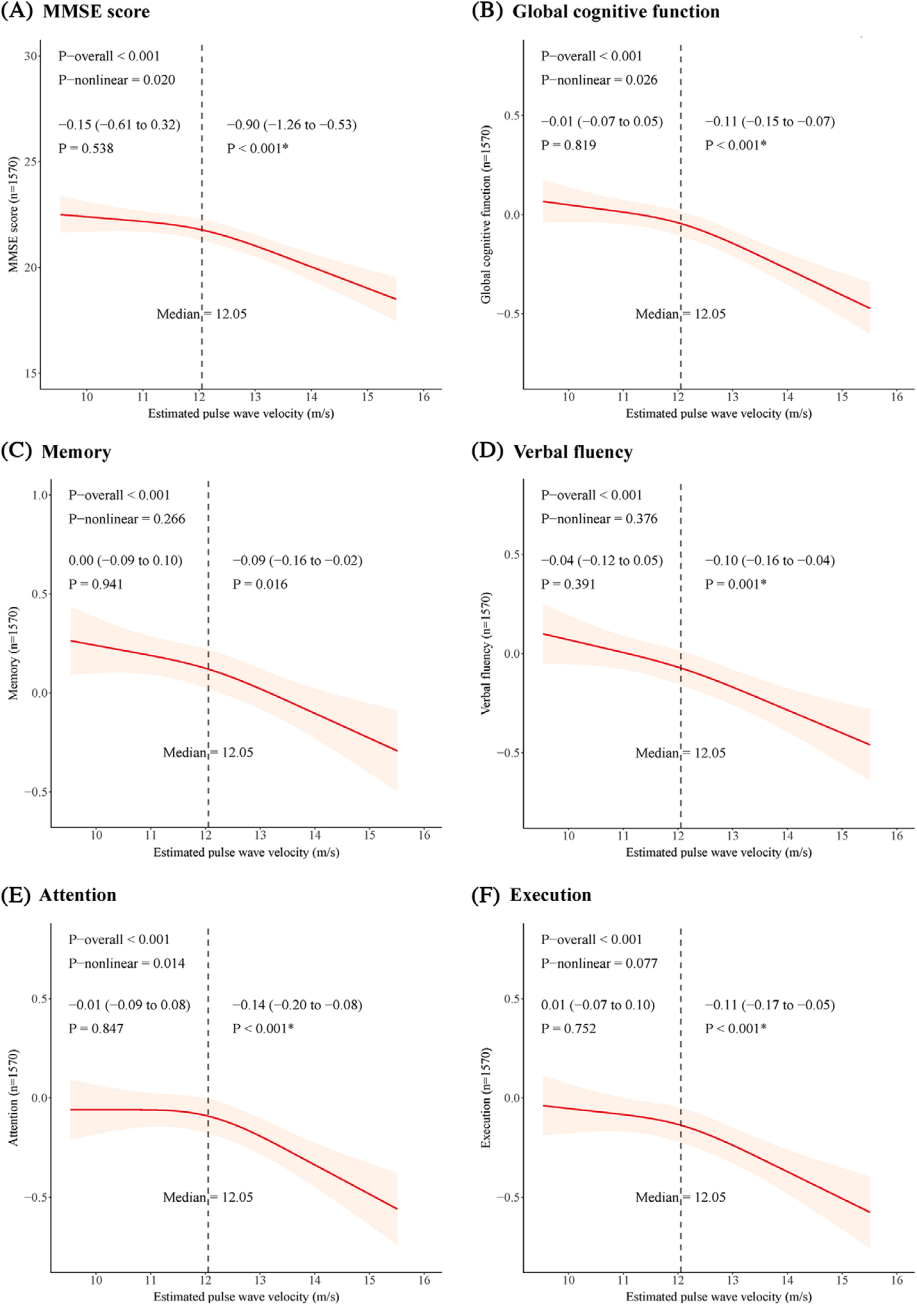
Associations of baseline ePWV with MMSE score and *z*‐scores of multiple cognitive domains assessed at the 4‐year follow‐up among dementia‐free participants at both baseline and follow‐up (*n* = 1570). (A) MMSE score; (B) global cognitive function; (C) memory; (D) verbal fluency; (E) attention; and (F) executive function. Solid lines represent MMSE score and adjusted *z*‐scores of cognitive domains, and shaded areas indicate 95% CIs derived from RCS regression models with three knots at the 10th, 50th, and 90th percentiles. The models were adjusted for sex, education, follow‐up time (in years), BMI, *APOE* genotype, smoking, alcohol consumption, use of antihypertensive medication, diabetes mellitus, hyperlipidemia, coronary heart disease, and stroke. BMI, body mass index; CI, confidence interval; ePWV, estimated pulse wave velocity; MMSE, Mini‐Mental State Examination; RCS, restricted cubic spline. ^*^The significant associations survived the Bonferroni corrections for multiple comparisons.

The RCS analysis indicated a nonlinear association of baseline ePWV with MMSE score and *z*‐scores of global cognition and attention assessed at follow‐up when controlling for all the examined covariates (all *p*‐overall < 0.05, *p*‐nonlinear < 0.05) (Figure 2A, B, and E). For the nonlinear associations, the inflection point was roughly aligned with the median of ePWV (i.e., 12.05 m/s). Then, we used the general linear regression models to further investigate the relationship of ePWV with MMSE score and *z*‐scores of global cognition and attention stratified by the median of the ePWV. For the purpose of comparison with the analysis of attention *z*‐score, we also used the general linear regression model to analyze the relationships of ePWV with other cognitive domains (i.e., memory, verbal fluency, and executive function) stratified by the median of ePWV, even though their nonlinear relationships were not statistically significant (Figure 2C, D, and F). Among individuals with ePWV ≥12.05 m/s, a higher ePWV at baseline was significantly associated with a lower MMSE score (*β* = −0.90; 95% CI = −1.26 to −0.53) and lower *z*‐scores of global cognition (*β* = −0.11; 95% CI = −0.15 to −0.07), memory (*β* = −0.09; 95% CI = −0.16 to −0.02), verbal fluency (*β* = −0.10; 95% CI = −0.16 to −0.04), attention (*β* = −0.14; 95% CI = −0.20 to −0.08), and executive function (*β* = −0.11; 95% CI = −0.17 to −0.05) assessed at follow‐up. Among persons with ePWV <12.05 m/s, no significant associations were observed between ePWV and any of the examined cognitive domains. After the Bonferroni correction, the association between ePWV and memory *z*‐score became statistically nonsignificant in individuals with ePWV ≥12.05 m/s (Figure 2).

## 4 DISCUSSION

In this population‐based prospective cohort study that targeted older adults who were living in rural communities in China, we found that greater ePWV was associated with an increased risk of incident dementia, AD, as well as a greater decline in global cognitive function. In addition, our study revealed a nonlinear association of baseline ePWV with MMSE score and *z*‐scores of global cognition and attention, but a linear association with memory, verbal fluency, and executive function assessed at the 4‐year follow‐up.

Previous studies have yielded mixed results regarding the association between arterial stiffness assessed with cfPWV and the risk of dementia.^12^, ^14^, ^36^, ^37^ For example, the 10‐year follow‐up data from the Framingham Offspring Study supported an association between higher cfPWV and an increased risk of incident dementia in people without diabetes.^12^ In addition, the Cardiovascular Health Study Cognition Study showed that higher cfPWV was associated with an increased risk of incident dementia.^14^ In contrast, both the Rotterdam study from the Netherlands and the Malmö Diet and Cancer study from Sweden found no association of cfPWV with incident dementia or subtypes of dementia.^36^, ^37^ Differences in the demographics of study populations and duration of follow‐up might partly contribute to the mixed findings across studies. A few population‐based studies have explored the relationship between arterial stiffness assessed with ePWV and dementia.^38^, ^39^ A cross‐sectional study of middle‐aged and older adults from the U.S. Health and Retirement Study (HRS) found that ePWV was independently associated with dementia.^38^ Furthermore, the post hoc analysis of Systolic Blood Pressure Intervention Trial Memory and Cognition in Decreased Hypertension (SPRINT‐MIND) found that a higher baseline ePWV was associated with an increased risk of probable dementia.^39^ These previous studies did not explore the relationship between ePWV and subtypes of dementia (AD and VaD). Our cohort study expanded the previous findings by showing the association between elevated ePWV and an increased risk of AD. Of note, the does–response association between higher levels of ePWV and the increased risk of VaD was less evident, largely due to limited statistical power because only 27 persons were diagnosed with incident VaD during the follow‐up period. In addition, attrition due to loss of follow‐up and competing risk of death might have resulted in underestimation of the true association between ePWV and risk of dementia (AD and VaD) because 422 persons who were lost to follow‐up (*n* = 287) or who died during the follow‐up (*n* = 135) had a higher ePWV than those individuals who were included in the study (ePWV: 12.98 vs 12.27 m/s, *p* < 0.001). Future long‐term large‐scale prospective cohort studies are warranted to verify the association between ePWV and incident dementia and subtypes of dementia in older adults.

Several population‐based studies have shown that increased cfPWV is associated with cognitive impairment and greater cognitive decline.^4^, ^10^ For instance, the UK Whitehall II Imaging Sub‐study showed that higher baseline cfPWV was closely related to poor semantic fluency and verbal learning at follow‐up.^4^ Data from the Toledo Study for Healthy Aging in Spain found that higher cfPWV was associated with worse cognitive performance and a greater cognitive decline in memory and executive function.^10^ Some studies have delved deeper into the association between ePWV and cognitive performance.^22^, ^40^ A cross‐sectional study of older Black and White adults from the National Health and Nutrition Examination Survey (NHANES) found that greater ePWV was associated with worse executive function.^40^ In addition, the Northern Manhattan Study suggested overall associations of greater ePWV with poor cognitive function and accelerated decline in global cognition, processing speed, episodic memory, executive function, and semantic memory.^22^ In line with these findings, our cohort study did link the higher ePWV with a greater decline in MMSE score and poorer performance on tests of multiple cognitive domains. Furthermore, our study revealed a nonlinear association of baseline ePWV with MMSE score and *z*‐scores of global cognition and attention at follow‐up, which has not yet been reported. Specifically, we identified an inflection point that corresponds to approximately the median of the ePWV, above which, a higher baseline ePWV was notably associated with a lower MMSE score and lower *z*‐scores of global cognition, memory, verbal fluency, attention, and executive function assessed at the 4‐year follow‐up independent of a range of potential confounders, below which no associations were observed between ePWV and any of the examined cognitive domains. These findings suggest that exposure to severe arterial stiffness may be an important determinant of subsequent cognitive dysfunction.

Several potential mechanisms may explain the associations of a greater ePWV with an elevated risk of dementia, AD, and accelerated cognitive decline. Arterial stiffening indicates a key hemodynamic process that contributes to microvascular brain injury by exposing cerebral small vasculature to high‐pressure fluctuations and flow pulsatility, which could further lead to cerebral small vessel diseases (CSVDs), such as white matter hyperintensities (WMHs), lacunar infarcts, and cerebral microbleeds; CSVDs have been associated with cognitive impairment and dementia.^41^, ^42^, ^43^ In addition, excessive flow pulsatility may contribute to accelerated atrophy in brain regions susceptible to AD (e.g., temporal lobe cortex and hippocampus). In particular, the blood supply to the hippocampus comes mainly from comparatively short branches originating from the circle of Willis, making it more susceptible to the detrimental impacts of abnormal central hemodynamics.^44^ Furthermore, increased arterial stiffness and the subsequent excessive pulsatility could lead to increased blood–brain‐barrier (BBB) permeability, neurovascular uncoupling, and reduced cerebral blood flow (CBF). As a result, the delivery of nutrients and the clearance of neurotoxic products (e.g., amyloid beta peptide) would be disrupted, thus potentially contributing to neurodegeneration and cognitive dysfunction.^45^

It is worth noting that the ePWV, estimated from age and MBP, does not capture all cardiovascular risks associated with arterial stiffness and cannot be a surrogate for measured cfPWV, although ePWV and cfPWV show similar predictive values for cardiovascular events.^17^ Previous studies have examined both cfPWV and ePWV in association with cognitive phenotypes.^6^, ^22^ However, compared with cfPWV, the ePWV is easier to acquire, and thus, more feasible for large‐scale population‐based studies as well as in routine clinical care. Future research may further evaluate the predictive value of ePWV against cfPWV for cognitive outcomes in old age.

The major strength of the current study refers to the population‐based cohort study that engaged rural‐dwelling Chinese older adults who received no or very limited school education and had low socioeconomic status, a sociodemographic group that has been substantially underrepresented in dementia research. Thus, findings from our study could help narrow the knowledge gap regarding the relevant risk factors of dementia and cognitive dysfunction in rural populations, and further help reduce rural health disparities in dementia.^46^ Some limitations of our study should be considered when interpreting the results. First, due to the relatively short follow‐up period, we could not rule out the potential reverse causality of the observed associations, especially given that dementia has up to over 20 years of the preclinical phase and that cardiovascular hemodynamics might be affected by cerebral neuropathology in the pre‐dementia phase.^47^, ^48^ Second, our study involved multiple outcomes of cognitive phenotypes. The statistical tests of multiple comparisons, although driven primarily by pre‐defined hypothesis, might increase the probability of false‐positive results. Indeed, some of the observed associations could not survive the corrections for multiple comparisons. Third, although we adjusted for a range of potential confounders in the analysis, residual confounding effects might still play a part owing to imperfect assessments of some confounders (e.g., self‐reported smoking and alcohol consumption) and lack of data on potentially relevant confounders (e.g., diet). Fourth, the study participants were recruited from only one rural region in western Shandong Province, which may limit the generalizability of the research findings to broader rural populations. Finally, we estimated ePWV from the equation that was derived from eight European populations, whether the equation is applicable to the Chinese population remained uncertain, although evidence has shown that ePWV is associated with cardiovascular events and all‐cause mortality in Chinese populations independently of demographic and cardiovascular risk factors.^49^

In summary, this community‐based cohort study of rural Chinese older adults revealed that higher ePWV was associated with an increased risk of dementia and AD, accelerated cognitive decline, and poorer performance in multiple cognitive domains. These findings highlight the potential that increased ePWV may be a risk factor for dementia and accelerated cognitive deterioration in this rural older population. Although the utility of ePWV in routine clinical practice remains to be defined by further clinical studies, from the public health perspective, ePWV can be an easily available tool for identifying individuals at risk for dementia and accelerated cognitive decline, thus offering the potential for early preventive and therapeutic (e.g., management of blood pressure) interventions to delay the onset of dementia.

## CONFLICT OF INTEREST STATEMENT

All authors have declared no conflicts of interest.

## CONSENT STATEMENT

All participants or informants in the SYS‐AD and the MIND‐China study provided written informed consent.

## Supporting information


Supporting Information
